# Editorial: Insights in digital health communication: 2023

**DOI:** 10.3389/fdgth.2024.1368666

**Published:** 2024-03-01

**Authors:** Carol Maher, Ben Singh

**Affiliations:** Alliance for Research in Exercise Nutrition and Activity (ARENA), University of South Australia, Adelaide, SA, Australia

**Keywords:** digital health communication, telehealth, health literacy, connected health, mhealth services

**Editorial on the Research Topic** Insights in digital health communication: 2023

Digital health communication plays an integral role in modern healthcare, from providing a conduit for spreading public health messages, to delivering health education, exchanging health data between patients and healthcare providers, and contributing to streamlined healthcare delivery. Such communications can leverage various technologies like telehealth platforms, mobile health apps, electronic health records, and social media. In doing so, digital health communication offers the potential to bridge geographical barriers, democratise access to health information, and foster a more collaborative and inclusive healthcare environment.

Yet, with the vast scope of digital health communication, the rapid pace of technology progression and evolving population needs, this field faces many challenges. The accessibility and quality of digital health tools can vary, leading to disparities in health information access. The need for interoperability among different digital health systems is crucial to ensure seamless communication and data exchange. Privacy and data security concerns are paramount and growing as sensitive health information is shared across digital platforms. Moreover, the digital divide—the gap between those with easy access to digital and information technology and those without—remains a significant hurdle. This Research Topic sets out to highlight the latest advancements in research across the digital health communication field, including new insights, novel developments, current challenges, and future perspectives in the field.

We feature four studies. The article *Improving health literacy using the power of digital communications to achieve better health outcomes for patients and practitioners,* by Fitzpatrick, reviews the role of digital communication tools in enhancing health literacy. It emphasises how mobile health apps, telemedicine, and online health information resources can promote better health outcomes by improving patient education, enabling self-management, and supporting clinical decision-making. The article also addresses the challenges of digital health literacy, such as access barriers, information reliability, and privacy concerns, and suggests strategies for overcoming these obstacles. In particular, it highlights the key international regulations and guidelines for protecting sensitive health data, ensuring transparent data governance, and maintaining individuals’ trust and confidence.

MacMahon and Richardson expand on integrating digital innovations into healthcare in a safe, regulated, and effective manner in their review: *Pathways, technology and the patient—connected health through the lifecycle*. They discuss the implementation of Connected Health, focusing on the standards and regulations vital for safe, effective, and secure health IT systems. Their review provides an overview of how technology changes healthcare pathways, emphasising the need for collaboration and communication in healthcare delivery organisations and manufacturers throughout the health IT system lifecycle. The paper also highlights the importance of Clinical Change Management in integrating technology into healthcare systems, and ensuring patient-centred care.

Two original studies are featured. *Analysis of Japanese consumers' attitudes toward the digital transformation of OTC medicine purchase behavior and eHealth literacy: an online survey for digital experience design*, by Tang et al., used an online survey to examine Japanese consumers' attitudes towards buying over-the-counter medicines in the context of increasing digitalisation. It explores their purchasing behaviours, preferences, and eHealth literacy, particularly in light of the COVID-19 pandemic which accelerated the digital transformation in healthcare. Findings reveal that Japanese consumers are seeking a combination of conventional and digital methods for purchasing OTC medicine. They prefer in-store purchases and instructions while also searching for additional decision-making information online. The findings suggest that a hybrid approach, that combines the reliability and personal touch of in-store experiences with the convenience and information-rich nature of digital platforms, may be optimal. This approach can enhance the overall consumer experience in purchasing OTC medicines while also potentially reducing risks associated with improper medication use.

Obegu et al., present insights from the implementation of a maternal and newborn mHealth service in Cameroon, in their study titled *Community participation for reproductive, maternal, newborn and child health: insights from the design and implementation of the BornFyne-prenatal management system digital platform in Cameroon*. This study focuses on stakeholder involvement in reproductive, maternal, newborn, and child health services. The article covers the methodology, including participatory research and stakeholder analysis. It discusses the results and challenges faced during the implementation, emphasising community engagement and digital health innovation in a low-middle income country context.

These studies underscore the transformative impact of digital health communication across diverse contexts and world regions. From enhancing health literacy and patient outcomes to navigating the nuances of consumer behavior in a digitally evolving landscape, they reveal the multifaceted nature of digital health communication. The studies highlight the need for a balanced approach that embraces technological innovation, while emphasising human-centric considerations. For instance, while the Japanese consumer study underscores a preference for a hybrid model in purchasing over-the-counter medicines, blending in-store experiences with online information, the research on the BornFyne-prenatal Management System in Cameroon emphasises the importance of community engagement and participatory approaches in digital health initiatives.

Furthermore, these studies collectively highlight the need for robust standards and regulations in the digital health domain. The articles suggest that the future of digital health communication lies not only in technological advancement but also in ensuring these advancements are accessible, reliable, and safeguard the privacy and security of users. The insights from these studies may be instrumental in guiding future digital health communication strategies, ensuring they are patient-centred, culturally sensitive, and inclusive, catering to the diverse needs of global populations.

